# LINC00839 knockdown restrains the metastatic behavior of nasopharyngeal carcinoma by sponging miR-454-3p

**DOI:** 10.18632/aging.203792

**Published:** 2021-12-29

**Authors:** Feng Ying Zhang, Xia Li, Ting Ting Huang, Mei Ling Xiang, Lin Lin Sun, Zhao Lan Sun

**Affiliations:** 1 Department of Otorhinolaryngology, Weifang Hospital of Traditional Chinese Medicine, Weifang, Shandong, China; 2 Department of Anesthesiology, Weifang Hospital of Traditional Chinese Medicine, Weifang, Shandong, China; 3 The First Clinical Medical College of Shandong University of Traditional Chinese Medicine, Jinan, Shandong, China

**Keywords:** nasopharyngeal carcinoma, invasion, EMT, LINC00839, miR-454-3p

## Abstract

Long intergenic non-coding RNA 00839 (LINC00839) has been verified as a pro-metastasis factor in malignancies. However, the significance of LINC00839 in nasopharyngeal carcinoma (NPC) has yet to be illuminated, as well as its underlying mechanism. Here, we disclosed that LINC00839 is highly expressed in NPC. Deletion of LINC00839 suppresses NPC cells rapid growth, invasive capacity and EMT *in vitro*. Besides, LINC00839 is identified as a “sponge” for miR-454-3p, and upregulation of LINC00839 reverses miR-454-3p-mediated inhibition of aggressiveness in NPC cells. Furthermore, the expression of cellular-mesenchymal epithelial transition factor (c-Met), the downstream target of miR-454-3p, is downregulated by LINC00839 knockdown in NPC cells. *In vivo*, LINC00839 knockdown retards the tumor growth of NPC cells in the xenografted mice model. Collectively, attenuation of LINC00839 expression attenuates the aggressive properties of NPC cells via directly sponging the miR-454-3p and regulating c-Met expression.

## INTRODUCTION

Nasopharyngeal carcinoma (NPC), which derives from nasopharyngeal epithelial cell, is a frequent subtype of head and neck cancer [1]. Due to patients with NPC usually with no specific symptoms in the early stage, most of them have stepped into advanced stages when diagnosed. As for the treatment, radiotherapy is the backbone of therapy for NPC and concurrent chemo-radiotherapy has become the therapeutic option for the advanced NPC [2]. Therefore, the five-year overall survival of patients with NPC has increased to about 70% [3]. Nevertheless, a complete cure of metastatic NPC remains elusive owing to its local recurrence and metastasis. The mechanism underlying NPC progression, including metastasis has not been completely understood yet.

Long intergenic non-coding RNAs (lincRNAs), more than 200 nucleotides in length, play a pivotal role in carcinogenesis and cancer progression [4]. As the competing endogenous RNAs (ceRNAs), lincRNAs competitively bind to miRNAs and thereby regulate miRNAs-targeted mRNAs [5]. In NPC, accumulated evidences have manifested that lincRNAs are implicated in tumorigenesis [6, 7]. For instance, lncRNA plasmacytoma variant translocation 1 (PVT1) modulates NPC cells growth by activating the lysine acetyltransferase 2A (KAT2A) acetyltransferase and stabilizing hypoxia-inducible factor-1alpha (HIF-1alpha) [4]. LncRNA Family With Sequence Similarity 225 Member A (FAM225A) facilitates NPC tumorigenesis and metastasis via sponging miR-590-3p/miR-1275 and upregulating β3 integrin (ITGB3) [1]. Conversely, several lncRNAs serve as anti-oncogenes and have profound prognostic significances in human NPC, such as lncRNA-LET, MEG3 and NKILA [8–10].

LINC00839 has been verified as a cancer-promoting factor in multiple malignancies, and its dysregulation is closely correlated with tumor initiation, development, and progression. In osteosarcoma, LINC00839 facilities the malignant development of cancer cell and dramatically elevates the expression of c-Met through competitively binding to miRNA-454-3p [11]. LINC00839 level is elevated in chemo-resistant breast carcinoma, and a higher level of LINC00839 is dramatically linked with inferior overall survival [12]. LINC00839 is elevated in neuroblastoma and identified to be significantly associated with survival [13]. To date, the roles of LINC00839 in malignancy of NPC have not been sufficiently elaborated.

In this study, we disclosed the tumor-promoting action of LINC00839 on the malignancy of NPC. A variety of functional experiments were implemented to illuminate the influence of LINC00839 knockdown on the growth, invasiveness and EMT of NPC cells *in vitro* and cells tumorigenicity *in vivo*. We validated that LINC00839 serves as a ceRNA for miR-454-3p and thereby positively regulates c-Met expression level.

## MATERIALS AND METHODS

### Cell lines

The nasopharyngeal epithelial cell line, NP-69 and NPC cells (SUNE-1, CNE-2, CNE-1 and C666-1) were cultured with RPMI-1640 supplemented with 10% FBS (Thermo Fisher Scientific, Waltham, MA, USA). C666-1 and SUNE-1 cells are not misidentification and contamination of human cell lines (ExPASy, https://www.expasy.org/). All cell lines were obtained from KeyGen Biotech Co., Ltd (Nanjing, Jiangsu, China) and maintained at 37°C in a humid chamber with 5% CO_2_.

### Cells transfection

MiR-454-3p mimic and its negative control (miR-NC), shRNA negative control (sh-Ctrl) and shRNA against LINC00839 (sh-LINC00839 #1 and sh-LINC00839 #2) were synthesized by GenePharma (Shanghai, China). The pcDNA3.1 plasmid (pc-vector) and plasmid pcDNA3.1-LINC00839 (pc-LINC00839) were purchased from GenePharma. pcDNA3.1 plasmid or miRNA mimic was transfected into C666-1 and SUNE-1 cells using Lipofectamine 3000 (Thermo Fisher Scientific).

### Quantitative real-time PCR analysis (qPCR)

Total RNAs in cells were extracted using a TRIZOL reagent kit (Thermo Fisher Scientific). RNA (1 μg) was used for cDNA synthesis with a reverse transcriptase kit (Thermo Fisher Scientific). qPCR was performed by using a SYBR Green qPCR Master Mix kit (TAKARA). The relative level of miR-454-3p was measured use an All-in-One miRNA qRT-PCR Detection Kit (GeneCopoeia, Rockville, Montgomery, USA). The primer sequences are shown below. miR-454-3p forward primer: 5′-ACCCTATCAATATTGTCTCTGC-3′, Reverse primer: 5′-GCGAGCACAGAATTAATACGAC-3′; U6 forward primer: 5′-AAAGCAAATCATCGGACGACC-3′, Reverse primer: 5′-GTACAACACATTGTTTCCTCGGA-3′; GAPDH forward primer: 5′-TGTGGGCATCAATGGATTTGG-3′, Reverse primer: 5′-ACACCATGTATTCCGGGTCAAT-3′; ZO-1 forward primer: 5′-GCCGCTAAGAGCACAGCAA-3′, Reverse primer: 5′-TCCCCACTCTGAAAATGAGGA-3′; Vimentin forward primer: 5′-GACGCCATCAACACCGAGTT-3′, Reverse primer: 5′-CTTTGTCGTTGGTTAGCTGGT-3′. Relative expression was calculated using the 2^−ΔΔCt^ method. U6 was used for miR-454-3p normalization and GAPDH was used for mRNA normalization.

### Cell growth

SUNE-1 or C666-1 cells (1 × 10^4^) were inoculated into 96 well culture plates. 20 μl of MTT (5 mg/ml) was added into plates at 1, 2, 3 or 4 days, respectively. Following the 4 h incubation, the supernatant in each well was gently removed and 200 μl of dimethyl sulfoxide (DMSO) solution was added into each well. The absorbance was determined at 450 nm.

### Clone-formation assay

SUNE1 or C666-1 cells (1 × 10^3^) were seeded into six-well plates. After two weeks, the cell colonies in plates were fixed with methanol and stained by 1% crystal violet (Sigma).

### Migration assay

After transfection, SUNE1 or C666-1 cells were seeded in 6-well plates. Upon reaching 80–90% confluency, a scratch was generated using a 100 μL pipette tip and cells were continually cultured for 24 h. The percentage of migration = (width at 0 h − width at 24 h)/width at 0 h × 100%.

### Cell invasion

The membrane in the Transwell chamber (8 μm pore size, Corning Costar, NY, USA) was coated with 40 μl of Matrigel (BD). 200 μl of SUNE1 or C666-1 cells were seeded into the upper chamber and the bottom chamber was filled with 500 μl of medium. After 24 h, the invading cells on the lower surface of membrane were dyed using 1% crystal violet and calculated.

### Immunoblotting

Total proteins were extracted from cells using the RIPA lysis buffer (Beyotime, Nanjing, China). 35 μg of proteins were separated using 10% SDS-PAGE and immediately transferred onto a PVDF membrane (Millipore, Braunschweig, Germany). Subsequently, the PVDF membrane was immunoblotted with c-Met, E-cadherin, N-cadherin or GAPDH antibody (1:1000, Santa Cruz Biotechnology, CA, USA) at 4°C overnight. After washing three times with TBST, the PVDF membrane was further incubated with HRP-linked secondary antibody (1:10000, Beyotime) for 2 h. The bands were assessed using an ECL kit (Millipore).

### Ago2-RNA immunoprecipitation (RIP) assay

After transfection of miR-454-3p, C666-1 and SUNE1 cell were collected, and cell lysate was prepared. Magna RIP™ RNA-binding protein immunoprecipitation kit (Millipore) was utilized to measure the mRNA level of LINC00839 bound to the IgG or Ago2 antibody. The retrieved RNAs were subject to qPCR analysis.

### RNA-fluorescence *in situ* hybridization (FISH)

FAM-labeled miR-454-3p probe and Cy3-labeled LINC00839 probe were obtained from RiboBio (Guangzhou, China). FISH assay was performed using a FISH kit (RiboBio) according to the manufacturer’s protocol. In brief, the probes were hybridized overnight. Then, cell nuclei were counterstained using DAPI (Beyotime). Fluorescence was measured using a fluorescence microscopy (Carl Zeiss, Germany).

### Luciferase reporter assay

The LINC00839 3′-UTR containing putative wild type binding sites for miR-454-3p was inserted into the pGL3 luciferase reporter (Promega, Madison, WI, USA) and named as pGL3-LINC00839-wt. The mutated binding sequence (pGL3-LINC00839-mut) was constructed by using a QuikChange Site-Directed Mutagenesis Kit (Stratagene, USA). C666-1 or SUNE1 cells were co-transfected with miR-454-3p plus pGL3-LINC00839-wt or miR-454-3p plus pGL3-LINC00839-mut. The c-Met 3′-UTR containing putative wild type binding sites or mutated binding sequence for miR-454-3p was inserted into pGL3 luciferase reporter to generate pGL3-c-Met-wt or pGL3-c-Met-mut, respectively. SUNE-1 or C666-1 cells were co-transfected with miR-454-3p plus pGL3-c-Met-wt or miR-454-3p plus pGL3-c-Met-mut. 48 h post transfection, the luciferase activities were detected utilizing a luciferase reporter assay kit (Promega).

### Tumor growth *in vivo*

BALB/c nude mice were subcutaneously inoculated with 100 μL of SUNE-1 (2 × 10^6^) cell suspension (*n* = 3 in each group). The length and width of xenograft tumors were recorded weekly. Tumor volume (mm^3^) = 0.5 × length × width^2^. 35 days after inoculation, the tumor-bearing mice were sacrificed. Tumor tissues were subject for immunohistochemical staining assay. The animal experiment was approved by the Weifang Hospital of traditional Chinese Medicine and performed according to the Guide for the Care and Use of Laboratory Animals.

### Statistical analysis

Data are presented as Mean ± SD. Statistical differences are assessed by Student’s *t*-test or one-way ANOVA analysis followed by Dunnett’s test. *P* value less than 0.05 is considered as statistical difference.

## RESULTS

### Knockdown of LINC00839 inhibits NPC cells growth *in vitro*

**Figure 1:**
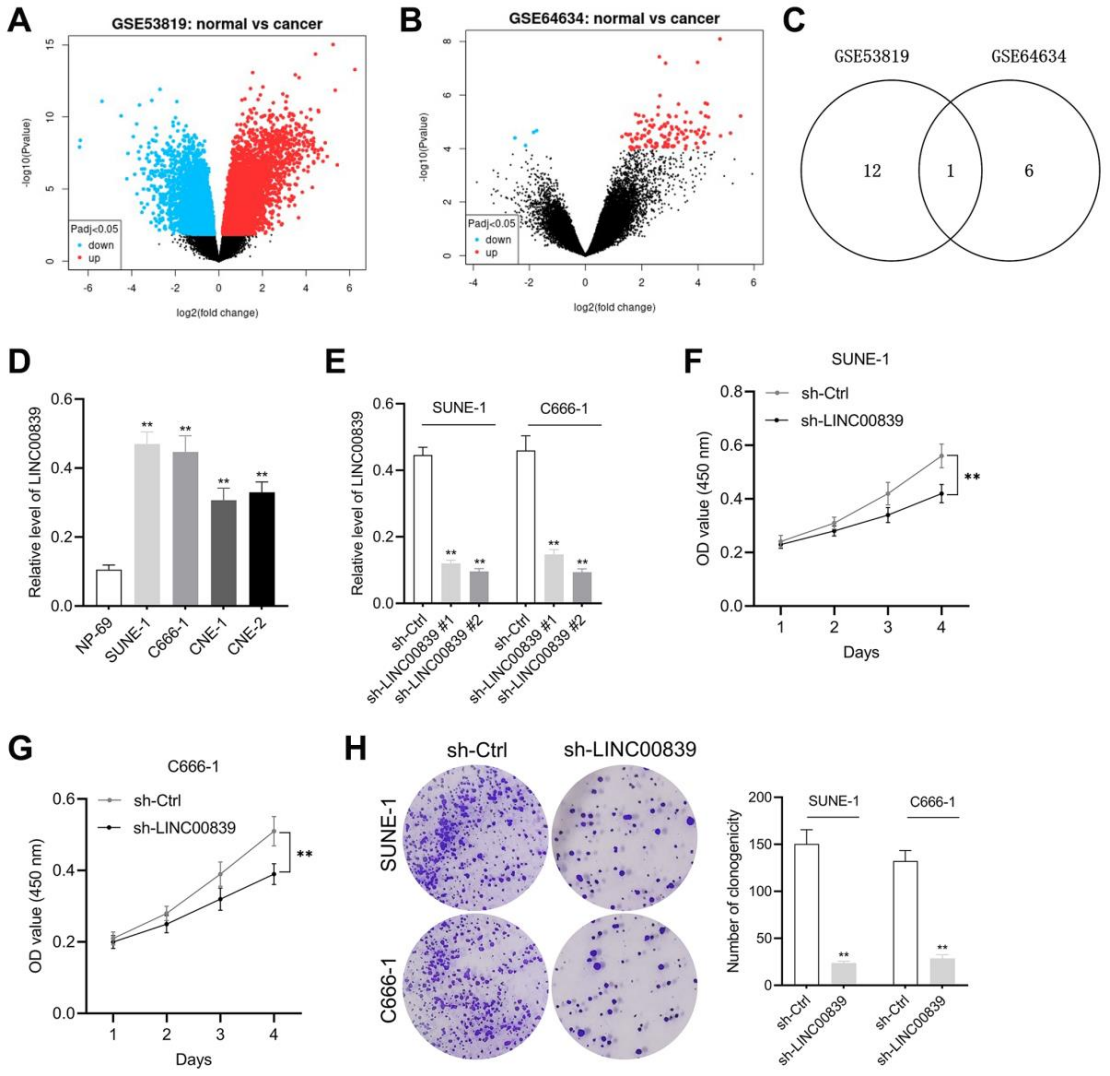
**The expression and effect of LINC00839 on cell growth in NPC.** (**A**, **B**) The differential gene expression in GSE53819 and GSE64634 data subsets was visualized using volcano plots. (**C**) Venn diagrams show the intersection of GSE53819 and GSE64634 data subsets. (**D**) qPCR examined LINC00839 expression in NPC cell lines (SUNE-1, CNE-1, C666-1 and CNE-2) compared with that in normal nasopharyngeal epidermal cell line NP-69. ^**^P < 0.01 compared with NP-69. (**E**) qPCR analysis testified LINC00839 expression in C666-1 and SUNE-1 cells after transfected with shRNA against LINC00839 (sh-LINC00839 # or sh-LINC00839 #2) or scrambled shRNA (sh-Ctrl). (**F**, **G**) After transfection, cell proliferation was determined by MTT assay. (**H**) The growth of NPC cells was determined by colony formation assay. ^**^P < 0.01 compared with sh-Ctrl group.

Two NPC-related GEO microarray chip data (GSE53819 and GSE64634) were screened for the identification of all differentially expressed genes (DEGs). The screening criteria by which the DEGs shown in the figure were identified were |log2 fold change|≥ 1 and *P*-value <0.05. Volcano plot displayed the DEGs in GSE53819 and GSE64634 (Figure 1A, 1B). Of these DEGs, total 342 differentially expressed lincRNAs in GSE53819 and 786 differentially expressed in GSE64634. According to the screening condition of fold change ≤ −1 and *P* < 0.05, 13 upregulated lincRNAs in GSE53819 and 7 upregulated lincRNAs in GSE64634 were obtained. Venn diagram was used to fine the intersection lincRNAs and LINC00839 was one of them (Figure 1C). To verify the expression pattern of LINC00839 in NPC, qPCR was performed to measure LINC00839 levels in NPC cell lines. As illustrated in Figure 1D, compared with normal cell line NP-69, LINC00839 expression was markedly elevated in NPC cell lines (SUNE-1, CNE-1, C666-1, and CNE-2). Then, SUNE-1 and C666-1 cells were transfected with sh-LINC00839 #1 or sh-LINC00839 #2. qPCR assay was performed to monitor the transfection efficiency (Figure 1E). We noted that sh-LINC00839 #2 exhibited higher shRNA transfection efficiency when compared with the sh-LINC00839 #1. Therefore, we chose to perform *in vitro* experiments with sh-LINC00839 #2 transfected cell line. In MTT assay, LINC00839 knockdown attenuated SUNE-1 and C666-1 cells proliferation (Figure 1F, 1G). Similarly, LINC00839 knockdown attenuated the clonogenic abilities of C666-1 and SUNE-1 cells *in vitro* (Figure 1H). These results demonstrate LINC00839 is upregulated in NPC and LINC00839 knockdown inhibits NPC cells growth *in vitro*.

### Knockdown of LINC00839 inhibits NPC cell EMT *in vitro*

**Figure 2:**
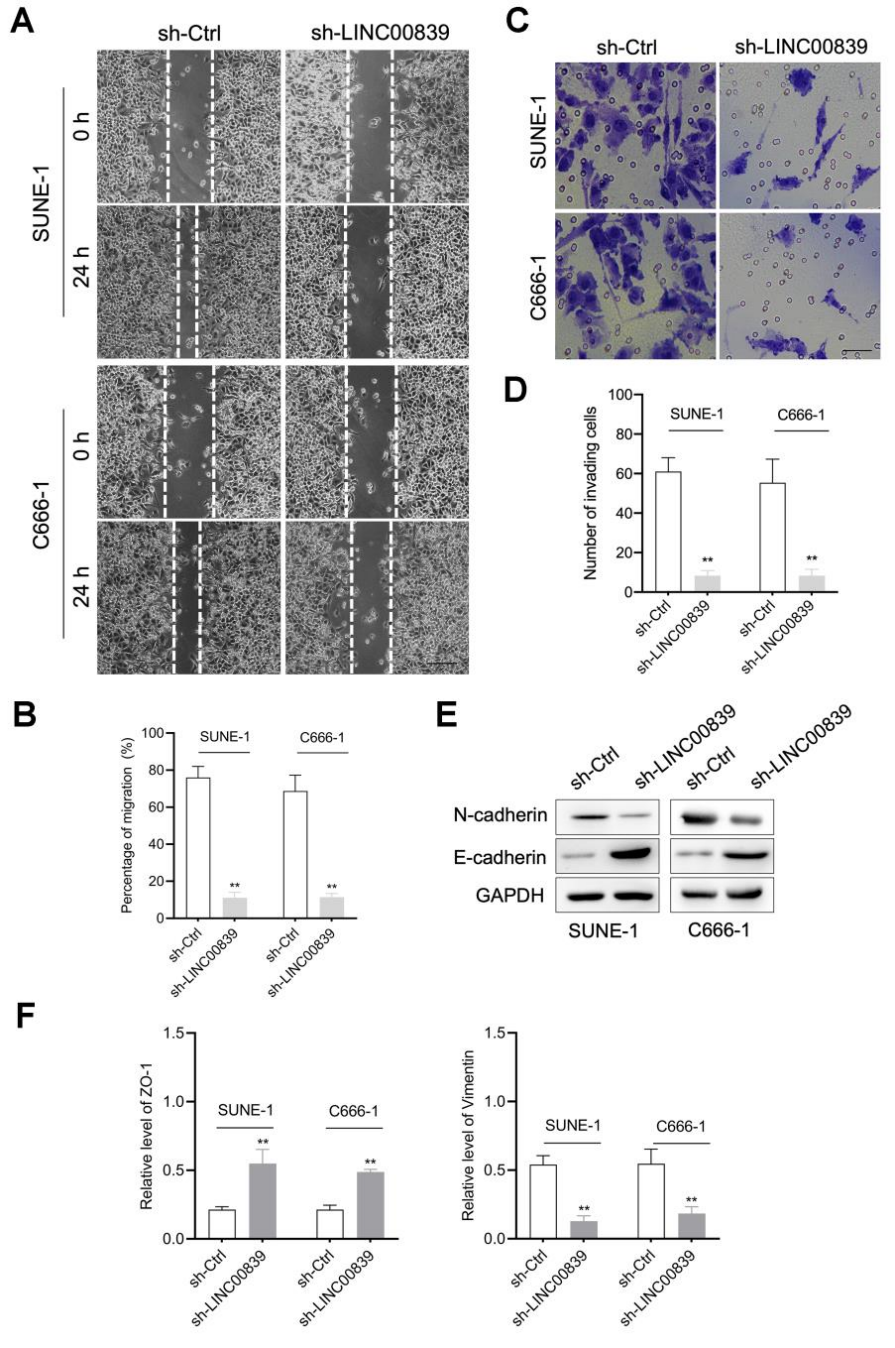
**The role of LINC00839 in NPC cell migration and invasion.** (**A**, **B**) After cell transfected with sh-LINC00839, cell migration was determined by wound healing assay. (**C**, **D**) Cell invasion was determined by Transwell assay. (**E**) The expressions of E-cadherin and N-cadherin were determined by western blot. (**F**) The mRNA levels of Vimentin and ZO-1 were measured by qPCR assay. ^**^P < 0.01 compared with sh-Ctrl group.

Besides, the capacities of cell migration, invasion and EMT were examined. Wound healing and Transwell assays showed C666-1 and SUNE-1 cell migration (Figure 2A, 2B) and invasive ability (Figure 2C, 2D) was attenuated in sh-LINC00839 group. Therefore, we further investigated the influence of LINC00839 knockdown on EMT in NPC cells *in vitro*. The result was that LINC00839 silencing in SUNE-1 and C666-1 cells caused level of N-cadherin and higher level of E-cadherin (Figure 2E). As illustrated in Figure 2F, knock-down of LINC00839 also decreased the mRNA of Vimentin (mesenchymal cell marker) and raised the level of ZO-1 (epithelial cell marker). These data suggested that silencing of LINC00839 diminishes the metastatic phenotypes of NPC cells.

### LINC00839 serves as a “sponge” for miR-454-3p

**Figure 3:**
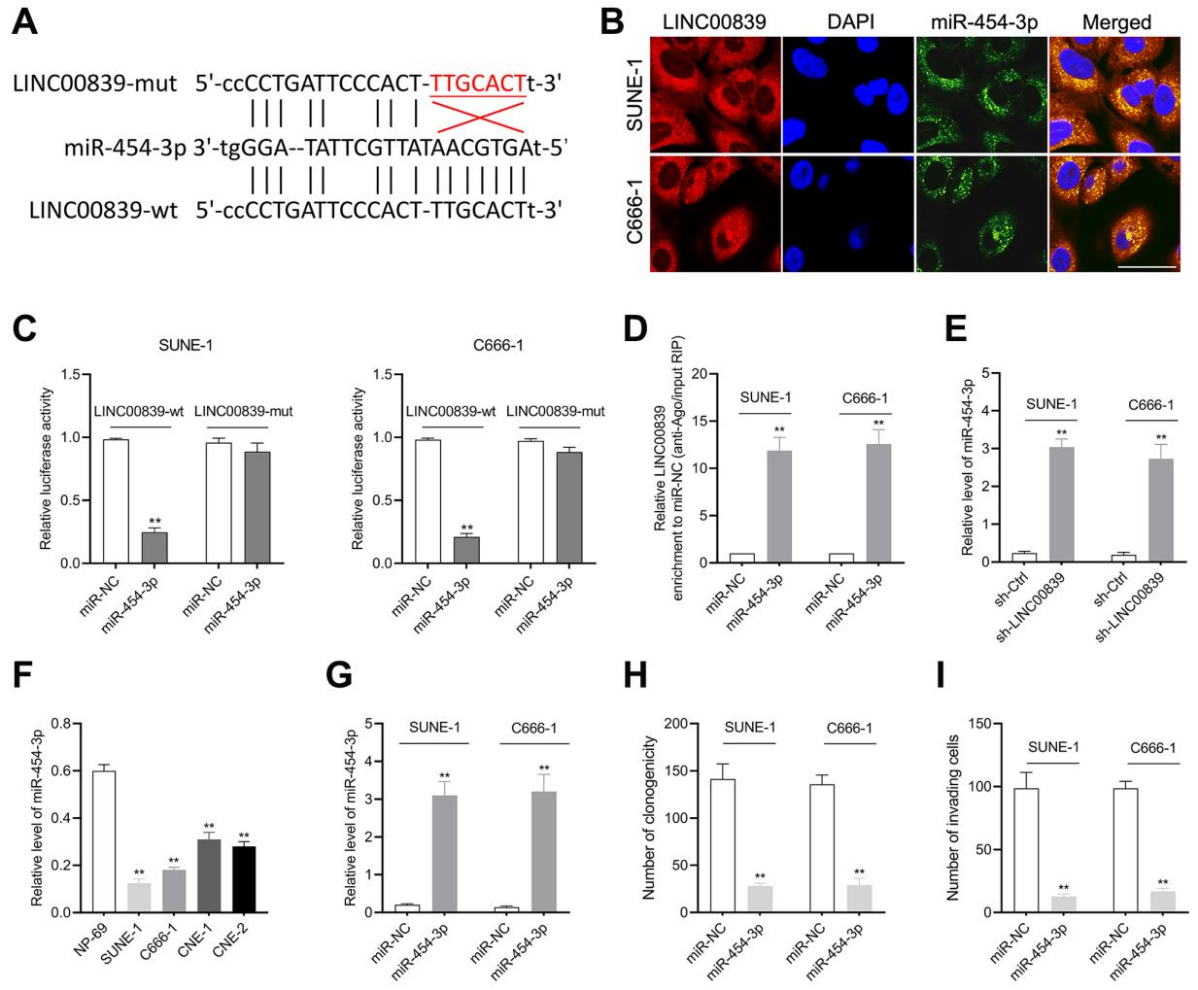
**The relationship between LINC00839 and miR-454-3p in SUNE-1 and C666-1 cells.** (**A**) Online software starBase showed the sequence alignment of miR-454-3p with the putative binding sites within LINC00839. (**B**) FISH analysis of the cellular colocalization of LINC00839 and miR-454-3p in SUNE-1 and C666-1 cell. Nuclei were stained with DAPI (scale bar, 20 μm). (**C**) The luciferase reporter assay demonstrated the influence of miR-454-3p on the luciferase activity in C666-1 and SUNE-1 cells transfected with LINC00839-wt or LINC00839-mut vector. (**D**) RIP assay was performed to further identify the potential binding of LINC00839 and miR-454-3p. (**E**) Levels of mIR-454-3p were detected by qPCR after cells transfected with sh-LINC00839. (**F**) qPCR examined miR-454-3p level in NPC cell lines (SUNE-1, CNE-1, C666-1 and CNE-2) compared with that in NP-69. ^**^P < 0.01 compared with NP-69. (**G**) qPCR analysis testified miR-454-3p expression in C666-1 and SUNE-1 cells after transfected with miR-NC or miR-454-3p mimic. (**H**) After transfection, the growth of NPC cells was determined by colony formation assay. (**I**) The invasion of NPC cells was analyzed using Transwell assay. ^**^P < 0.01 compared with miR-NC group.

With the research on online bioinformatics database (starBase v2.0), we found miR-454-3p was predicted to have complementary base pairing with LINC00839 (Figure 3A). Subsequently, the FISH assay was carried out, and the results displayed that miR-454-3p and LINC00839 were colocalized in the cytoplasm of SUNE-1 and C666-1 cells (Figure 3B). To verify the target binding of miR-454-3p and LINC00839, we constructed luciferase reporter vectors containing the predicted wild-type (wt) or mutant (mut) binding sites of miR-454-3p on LINC00839 (named as LINC00839-wt and LINC00839-mut). As shown in Figure 3C, the luciferase activity was declined in NPC cells co-transfected with miR-454-3p mimic and LINC00839-wt. Ago2-RIP assay further showed LINC00839 enrichment was more pronounced in miR-454-3p overexpression group than that in the miR-NC group (Figure 3D). Moreover, LINC00839 silencing remarkedly increased the level of miR-454-3p (Figure 3E). In comparison with NP-69 cell, miR-454-3p was highly expressed in NPC cells as demonstrated by qPCR analysis (Figure 3F). SUNE-1 and C666-1 cells were transduced with miR-NC or miR-454-3p mimic (miR-454-3p). As shown in Figure 3G, miR-454-3p was dramatically upregulated in C666-1 and SUNE-1 cells. Subsequently, we observed that miR-454-3p mimic decreased the clone formation capacity in NPC cells (Figure 3H). In terms of the capacity of cell invasion, Transwell assays showed that invasive cells were declined in miR-454-3p-overexpressed NPC cells (Figure 3I). Collectively, these findings successfully suggest that miR-454-3p is downregulated in NPC and exerts the tumor suppressor role in NPC.

### c-Met is targeted by miR-454-3p

**Figure 4:**
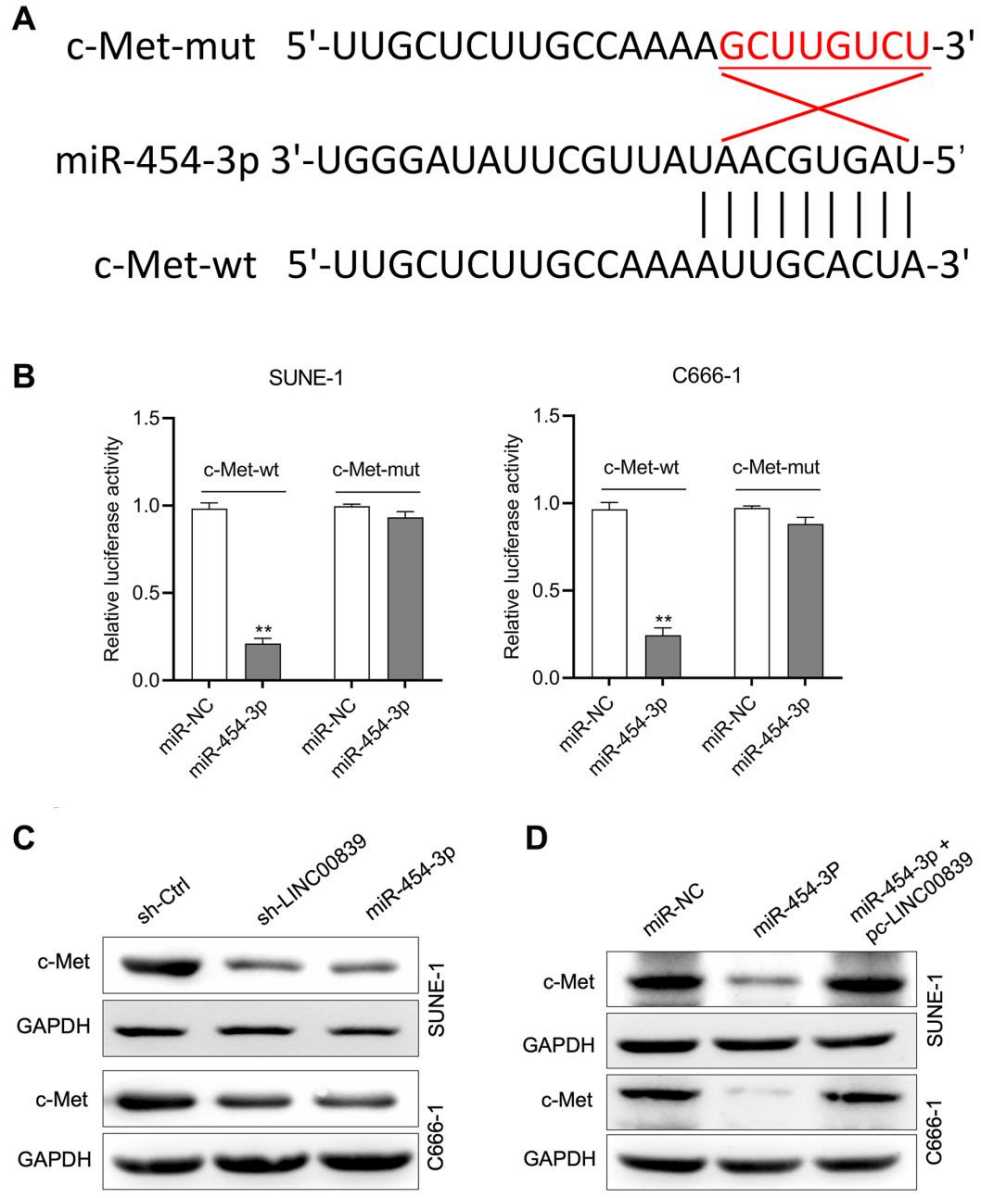
**c-Met was a target gene for miR-454-3p.** (**A**) The predicted miR-454-3p binding sites in the 3′-UTR of c-Met gene according to Targetscan. (**B**) Luciferase activity of wild-type (wt) or mutant (mut) c-Met 3′-UTR in SUNE-1 and C666-1 cells transfected with miR-454-3p mimic or miR-NC. ^**^P < 0.01 compared with miR-NC group. (**C**) Western blot detected the effect of miR-454-3p and sh-LINC00839 on c-Met protein expression in SUNE-1 and C666-1 cells. (**D**) SUNE-1 and C666-1 cells were transfected with miR-454-3p or co-transfected with miR-454-3p and pc-LINC00839. The expression of c-Met was determined by western blot.

According to in silico data on TargetScan Human (TargetScan Human release 7.2), the putative target genes of miR-454-3p were investigated and bioinformatics analysis indicated that human c-Met was a potential target of miR-454-3p (Figure 4A). To confirm this prediction, c-Met 3′-UTR wild type (c-Met-wt) and mutant (c-Met-mut) reporter plasmids were constructed, and the luciferase reporter gene test was conducted. The luciferase activity in cells transfected with c-Met-wt rather than c-Met-mut was remarkably reduced by miR-454-3p mimic (Figure 4B). Additionally, the regulatory effect of LINC00839 and miR-454-3p on c-Met expression was determined by immunoblotting. The result showed that c-Met expression was inhibited when cells were transfected with miR-454-3p or sh-LINC00839 (Figure 4C). In co-transfection group, re-introducing LINC00839 (pc-LINC00839) almost restored the expression level of c-Met in miR-454-3p mimic transfected NPC cells (Figure 4D). Altogether, these findings demonstrate that LINC00839 serves as a “sponge” through competitively binding to the endogenous miR-454-3p.

### LINC00839 overexpression abates the effects of miR-454-3p

**Figure 5:**
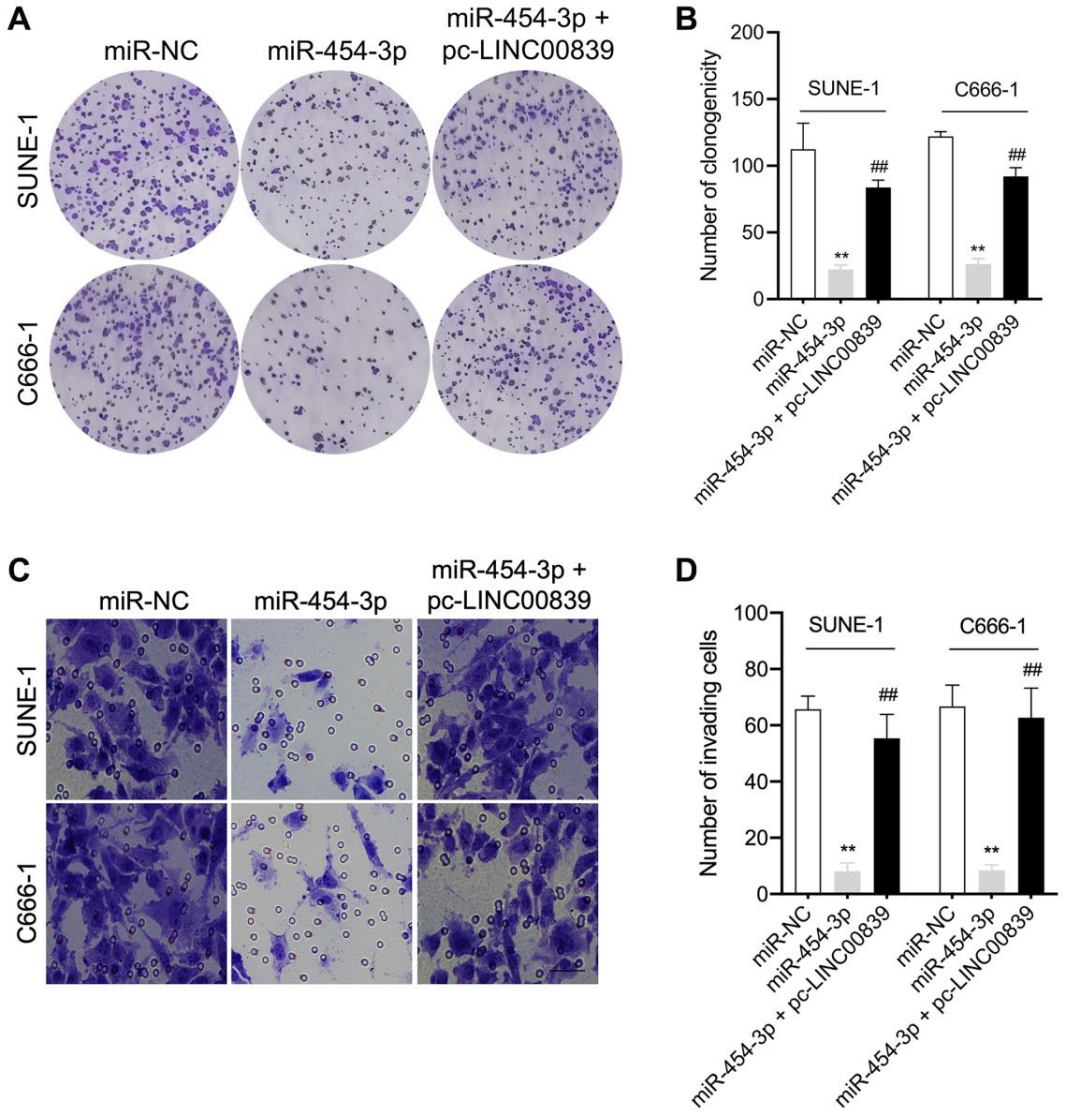
**LINC00839 reverses the effects of miR-454-3p in SUNE-1 and C666-1 cells.** (**A**–**B**) SUNE-1 and C666-1 cells were transfected with miR-454-3p or co-transfected with miR-454-3p and pc-LINC00839. The growth of NPC cells was measured using colony formation assay. (**C**–**D**) The invasion of NPC cells was assessed using Transwell assay. ^**^P < 0.01 compared with miR-NC group, ^##^*P* < 0.01 compared with miR-454-3p group.

To further explore the mechanism of LINC00839 in affecting the malignancy of NPC, the rescue experiments using pcDNA3.1-LINC00839 plasmid (pc-LINC00839) were performed to ascertain the role of LINC00839 in mediating the biological action of miR-454-3p in NPC cells *in vitro*. SUNE-1 and C666-1 cells co-transfected with pc-LINC00839 and miR-454-3p. Transfection cells were divided into three groups: miR-NC, miR-454-3p mimic and pc-LINC00839 + miR-454-3p (Figure 4D). In miR-454-3p mimic group, cells exhibited lower cell colony formation, which was rescued by upregulation of LINC00839 (Figure 5A, 5B). Cell invasion ability was rescued in pc-LINC00839 + miR-454-3p group, compared to cells treated with miR-454-3p mimic (Figure 5C, 5D). These observations reveal a novel LINC00839/miR-454-3p axis in NPC development.

### LINC00839 knockdown alleviates NPC cells growth *in vivo*

**Figure 6:**
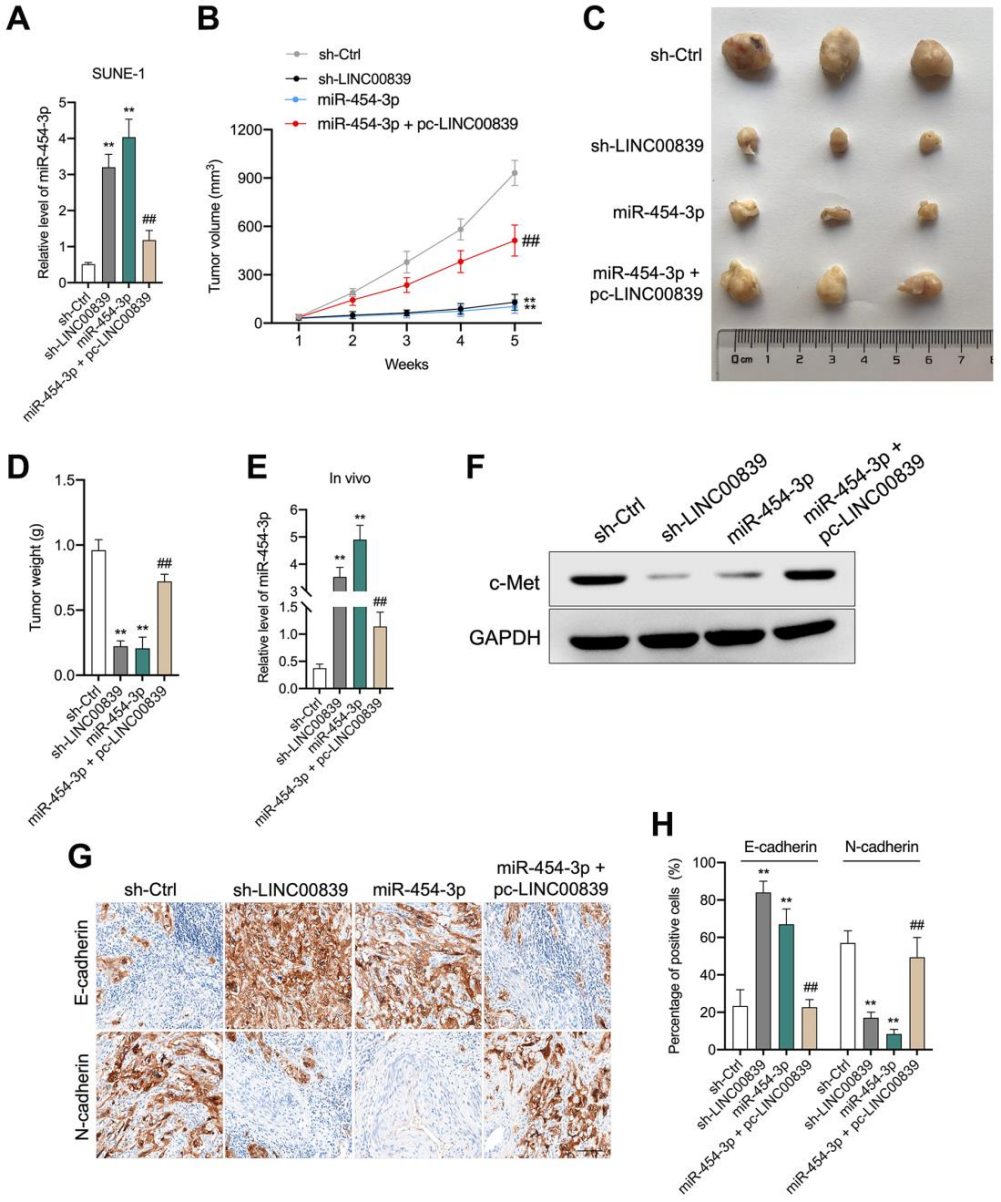
**LINC00839 knockdown suppresses tumor growth *in vivo*.** (**A**) SUNE-1 cells were transected with sh-LINC00839, or miR-454-3p mimic or co-transfected with miR-454-3p mimic and pc-LINC00839, the level was miR-454-3p was assessed by qPCR. (**B**) SNUE-1 cells were xenogenic transplanted into nude mice (*n* = 3). Tumor volumes were measured every week and the growth curve of xenograft tumors was drawn. (**C**) Representative images of xenograft tumors. (**D**) Tumor weight. (**E**) Expression levels of miR-454-3p in xenograft tumors were determined by qPCR. (**F**) Expression level of c-Met in xenograft tumors was determined by western blot. (**G**, **H**) Protein levels of E-cadherin and N-cadherin were examined by immunohistochemical staining. ^**^P < 0.01 compared with sh-Ctrl group, ^##^*P* < 0.01 compared with miR-454-3p group.

Our *in vitro* findings corroborated that LINC00839 act as a “ceRNA” for miR-454-3p. To confirm our results *in vivo*, a nude mice xenograft experiment was implemented. SUNE-1 cells were transfected with sh-LINC00839 or miR-454-3p mimic and then were subcutaneously inoculated into nude mice (Figure 6A–6C). Another group of mice was inoculated with miR-454-3p mimic + pc-LINC00839 co-transfected SUNE-1 cells. After 5 weeks, compared with the sh-Ctrl group, the tumor growth rate was inhibited in sh-LINC00839 group and miR-454-3p group. When compared with the miR-454-3p group, SUNE-1 cells growth *in vivo* was restored in mice injected with miR-454-3p mimic + pc-LINC00839 co-transfected cells (Figure 6B, 6C). Similarly, the weight of xenograft tumors also showed the same trend (Figure 6D). The level of miR-454-3p was raised in sh-LINC00839 group and miR-454-3p group. The level of miR-454-3p increased by miR-454-3p was counteracted in tumor tissue formed by miR-454-3p + pc-LINC00839 co-transfected SUNE-1 cells (Figure 6E). Furthermore, c-Met expression was extremely downregulated in sh-LINC00839 group and miR-454-3p group. The expression of c-Met decreased by miR-454-3p was restored in mice inoculated with miR-454-3p + pc-LINC00839 co-transfected SUNE-1 cells (Figure 6F). Besides, the expression of N-cadherin was abundantly attenuated by sh-LINC00839 and miR-454-3p transfection whereas the expression of E-cadherin was raised as demonstrated by immunohistochemical staining (Figure 6G, 6H). When compared with those in the miR-454-3p group, the protein expression patterns of E-cadherin and N-cadherin were reversed by pc-LINC00839. These data demonstrated the tumor-suppressive role of LINC00839 knockdown in NPC tumor growth.

## DISCUSSION

Despite the advancements in molecularly targeted approaches in NPC treatment, the 5-year survival rate of patients is still not optimistic owing to distant metastases [14]. This triggers a major effort to discover actionable molecular targets to treat patients with NPC. Recently, an extensive body of lncRNAs has been demonstrated as playing pivotal roles in the pathogenesis of NPC [7].

LINC00839 acts as an oncogenic gene and is significantly upregulated in human cancers including breast cancer and osteosarcoma [11, 12]. In our study, we noticed that LINC00839 level is widely higher in NPC tissues from GEO dataset and NPC cell lines than the normal tissues and NP-69. Here, we figured out the role of LINC00839 knockdown on the malignancy of NPC in C666-1 and SUNE-1 cells. The results suggested that knockdown of LINC00839 abated the malignancy of NPC cells *in vitro* through inhibiting cell proliferation, colony formation, invasion and EMT. The tumorigenicity of NPC cells *in vivo* was also retarded by LINC00839 knockdown. Taken all results together, LINC00839 is upregulated in human NPC and its dysregulation expression modulates the initiation and development of NPC.

Specific endogenous lincRNAs contain miRNAs binding sites and function as “sponge” for specific miRNAs, thus regulate gene expression [1, 15]. For instance, lncRNA CCDC144NL-AS1 facilitates the oncogenicity of osteosarcoma via acting as “sponge” for miRNA-490-3p and increasing high mobility group AT-Hook 2 (HMGA2) expression [16]. In this study, we figured out LINC00839 function as “sponge” for miR-454-3p. It is reviewed that miR-454-3p is expressed in various human tissues under normal physiological conditions and pathologic conditions; it is implicated in a range of pathologic processes, for example cancerous cell proliferation, metastasis and EMT [17]. Although deregulated miRNAs including miR-454-3p is yet fully understood, miRNAs have been recognized as new diagnostic and prognostic biomarkers in NPC, as well as the therapeutic targets. Some researchers indicated that combined miRNA and current chemotherapeutic drugs could induce some promising results *in vitro*. We observed that miR-454-3p overexpression suppress NPC cells proliferation and invasion, as well as EMT.

In ceRNA network, the function of lincRNAs depends on the miRNA target. With the assistance of a computational prediction tool, we identified c-Met as a possible target gene of miR-454-3p, which was corroborated by the western blot and luciferase reporter assay. c-Met, a member of the ADAM family, is frequently overexpressed in breast, ovarian, and prostate cancers [18]. A close association of c-Met expression with cervical lymph node metastasis in NPC has been disclosed [19]. Silencing of c-Met diminishes the proliferation and invasion abilities of NPC cells [20]. Additionally, upregulation of LINC00839 significantly reversed miR-454-3p-induced promoting role in NPC cells *in vitro*, implying a novel LINC00839/miR-454-3p signaling axis in NPC development. Consistent with the observations *in vitro*, LINC00839 knockdown alleviated the growth of NPC cells and acted as a ceRNA to prevent miR-454-3p from inhibiting its target gene c-Met *in vivo*.

To sum up, we showed the knockdown of LINC00839 restrains the growth, aggressive traits and EMT process in NPC cells C666-1 and SUNE-1 through acting as the miR-454-3p sponge and downregulating c-Met. Our research is of considerable value in terms of NPC diagnosis and prognosis, and provides new biomarkers for the targeted therapy in NPC.

## References

[1] Zheng ZQ, Li ZX, Zhou GQ, Lin L, Zhang LL, Lv JW, Huang XD, Liu RQ, Chen F, He XJ, Kou J, Zhang J, Wen X, et al. Long Noncoding RNA FAM225A Promotes Nasopharyngeal Carcinoma Tumorigenesis and Metastasis by Acting as ceRNA to Sponge miR-590-3p/miR-1275 and Upregulate ITGB3. Cancer Res. 2019; 79:4612–26. 10.1158/0008-5472.CAN-19-0799 31331909

[2] Wen X, Liu X, Mao YP, Yang XJ, Wang YQ, Zhang PP, Lei Y, Hong XH, He QM, Ma J, Liu N, Li YQ. Long non-coding RNA DANCR stabilizes HIF-1α and promotes metastasis by interacting with NF90/NF45 complex in nasopharyngeal carcinoma. Theranostics. 2018; 8:5676–89. 10.7150/thno.28538 30555573 PMC6276287

[3] Zhang B, Hu Y, Xiong RH, Pan YF, Xu QL, Kong XY, Cai R, Chen QQ, Tang HY, Jiang W. Matched analysis of induction chemotherapy plus chemoradiotherapy versus induction chemotherapy plus radiotherapy alone in locoregionally advanced nasopharyngeal carcinoma: a multicenter study. Oncotarget. 2017; 8:14078–88. 10.18632/oncotarget.13285 27845907 PMC5355164

[4] Wang Y, Chen W, Lian J, Zhang H, Yu B, Zhang M, Wei F, Wu J, Jiang J, Jia Y, Mo F, Zhang S, Liang X, et al. The lncRNA PVT1 regulates nasopharyngeal carcinoma cell proliferation via activating the KAT2A acetyltransferase and stabilizing HIF-1α. Cell Death Differ. 2020; 27:695–710. 10.1038/s41418-019-0381-y 31320749 PMC7206084

[5] Luan W, Ding Y, Yuan H, Ma S, Ruan H, Wang J, Lu F, Bu X. Long non-coding RNA LINC00520 promotes the proliferation and metastasis of malignant melanoma by inducing the miR-125b-5p/EIF5A2 axis. J Exp Clin Cancer Res. 2020; 39:96. 10.1186/s13046-020-01599-7 32466797 PMC7254730

[6] Zheng YJ, Zhao JY, Liang TS, Wang P, Wang J, Yang DK, Liu ZS. Long noncoding RNA SMAD5-AS1 acts as a microRNA-106a-5p sponge to promote epithelial mesenchymal transition in nasopharyngeal carcinoma. FASEB J. 2019; 33:12915–28. 10.1096/fj.201900803R 31557058 PMC6902713

[7] Hu W, Li H, Wang S. LncRNA SNHG7 promotes the proliferation of nasopharyngeal carcinoma by miR-514a-5p/ELAVL1 axis. BMC Cancer. 2020; 20:376. 10.1186/s12885-020-06775-8 32370736 PMC7202000

[8] Chak WP, Lung RW, Tong JH, Chan SY, Lun SW, Tsao SW, Lo KW, To KF. Downregulation of long non-coding RNA MEG3 in nasopharyngeal carcinoma. Mol Carcinog. 2017; 56:1041–54. 10.1002/mc.22569 27597634

[9] Sun Q, Liu H, Li L, Zhang S, Liu K, Liu Y, Yang C. Long noncoding RNA-LET, which is repressed by EZH2, inhibits cell proliferation and induces apoptosis of nasopharyngeal carcinoma cell. Med Oncol. 2015; 32:226. 10.1007/s12032-015-0673-0 26243049

[10] Zhang W, Guo Q, Liu G, Zheng F, Chen J, Huang D, Ding L, Yang X, Song E, Xiang Y, Yao H. NKILA represses nasopharyngeal carcinoma carcinogenesis and metastasis by NF-κB pathway inhibition. PLoS Genet. 2019; 15:e1008325. 10.1371/journal.pgen.1008325 31430288 PMC6716677

[11] Zhang Y, Guo H, Ma L, Chen X, Chen G. Long Noncoding RNA LINC00839 Promotes the Malignant Progression of Osteosarcoma by Competitively Binding to MicroRNA-454-3p and Consequently Increasing c-Met Expression. Cancer Manag Res. 2020; 12:8975–87. 10.2147/CMAR.S269774 33061593 PMC7522415

[12] Chen Q, Shen H, Zhu X, Liu Y, Yang H, Chen H, Xiong S, Chi H, Xu W. A nuclear lncRNA Linc00839 as a Myc target to promote breast cancer chemoresistance via PI3K/AKT signaling pathway. Cancer Sci. 2020; 111:3279–91. 10.1111/cas.14555 32619088 PMC7469761

[13] Meng X, Fang E, Zhao X, Feng J. Identification of prognostic long noncoding RNAs associated with spontaneous regression of neuroblastoma. Cancer Med. 2020; 9:3800–15. 10.1002/cam4.3022 32216054 PMC7286466

[14] Lee HM, Okuda KS, González FE, Patel V. Current Perspectives on Nasopharyngeal Carcinoma. Adv Exp Med Biol. 2019; 1164:11–34. 10.1007/978-3-030-22254-3_2 31576537

[15] Kong YG, Cui M, Chen SM, Xu Y, Xu Y, Tao ZZ. LncRNA-LINC00460 facilitates nasopharyngeal carcinoma tumorigenesis through sponging miR-149-5p to up-regulate IL6. Gene. 2018; 639:77–84. 10.1016/j.gene.2017.10.006 28987345

[16] He J, Guan J, Liao S, Wu Z, Liu B, Mo H, Yuan Z. Long Noncoding RNA CCDC144NL-AS1 Promotes the Oncogenicity of Osteosarcoma by Acting as a Molecular Sponge for microRNA-490-3p and Thereby Increasing HMGA2 Expression. Onco Targets Ther. 2021; 14:1–13. 10.2147/OTT.S280912 33442262 PMC7797336

[17] Zuo J, Yu H, Xie P, Liu W, Wang K, Ni H. miR-454-3p exerts tumor-suppressive functions by down-regulation of NFATc2 in glioblastoma. Gene. 2019; 710:233–9. 10.1016/j.gene.2019.06.008 31181312

[18] Huang L, Xie K, Li H, Wang R, Xu X, Chen K, Gu H, Fang J. Suppression of c-Met-Overexpressing Tumors by a Novel c-Met/CD3 Bispecific Antibody. Drug Des Devel Ther. 2020; 14:3201–14. 10.2147/DDDT.S254117 32982167 PMC7495354

[19] Horikawa T, Sheen TS, Takeshita H, Sato H, Furukawa M, Yoshizaki T. Induction of c-Met proto-oncogene by Epstein-Barr virus latent membrane protein-1 and the correlation with cervical lymph node metastasis of nasopharyngeal carcinoma. Am J Pathol. 2001; 159:27–33. 10.1016/S0002-9440(10)61669-0 11438450 PMC1850422

[20] Li Y, Zhang S, Tang Z, Chen J, Kong W. Silencing of c-Met by RNA interference inhibits the survival, proliferation, and invasion of nasopharyngeal carcinoma cells. Tumour Biol. 2011; 32:1217–24. 10.1007/s13277-011-0225-y 21922276

